# Prerequisites for cytokine measurements in clinical trials with multiplex immunoassays

**DOI:** 10.1186/1471-2172-10-52

**Published:** 2009-09-28

**Authors:** Wilco de Jager, Katarzyna Bourcier, Ger T Rijkers, Berent J Prakken, Vicki Seyfert-Margolis

**Affiliations:** 1 Department of Pediatric Immunology, Centre for Molecular and Cellular Intervention (CMCI), University Medical Centre Utrecht, Utrecht, The Netherlands; 2 EUREKA Institute of Translational Medicine, Siracusa, Italy; 3 Immune Tolerance Network, Luminex Core Facility, Utrecht, the Netherlands; 4 Immune Tolerance Network, University of California, San Francisco, CA, Bethesda, MD, USA; 5 Laboratory of Medical Microbiology and Immunology, Sint Antonius Hospital, Nieuwegein, the Netherlands

## Abstract

**Background:**

Growing knowledge about cellular interactions in the immune system, including the central role of cytokine networks, has lead to new treatments using monoclonal antibodies that block specific components of the immune system. Systemic cytokine concentrations can serve as surrogate outcome parameters of these interventions to study inflammatory pathways operative in patients *in vivo*. This is now possible due to novel technologies such as multiplex immunoassays (MIA) that allows detection of multiple cytokines in a single sample. However, apparently trivial underappreciated processes, (sample handling and storage, interference of endogenous plasma proteins) can greatly impact the reliability and reproducibility of cytokine detection.

Therefore we set out to investigate several processes that might impact cytokine profiles such as blood collecting tubes, duration of storage, and number of freeze thawing cycles.

**Results:**

Since under physiological conditions cytokine concentrations normally are low or undetectable we spiked cytokines in the various plasma and serum samples. Overall recoveries ranged between 80-120%. Long time storage showed cytokines are stable for a period up to 2 years of storage at -80°C. After 4 years several cytokines (IL-1α, IL-1β, IL-10, IL-15 and CXCL8) degraded up to 75% or less of baseline values. Furthermore we show that only 2 out of 15 cytokines remained stable after several freeze-thawing cycles. We also demonstrate implementation of an internal control for multiplex cytokine immunoassays.

**Conclusion:**

All together we show parameters which are essential for measurement of cytokines in the context of clinical trials.

## Background

Better characterization of cellular processes and cytokine pathways in a variety of diseases ranging from allergy and autoimmunity to cancer has lead to new treatments that use monoclonal antibodies which specifically block components of the human immune system including cytokine pathways [1-6]. These new therapeutic strategies, which modulate inflammatory processes of the immune system, can induce major changes in the downstream cytokine milieu. Indeed, the aftermath of the TGN1412 phase I clinical trial in March 2006 revealed that the life threatening adverse events were the consequence of a rapid onset severe cytokine storm [7,8]. This example underscores the importance of monitoring cytokines during experimental therapies which are based on or could influence cytokine pathways or cytokine producing cells.

Cytokines are small secreted extra-cellular signaling (glyco-) proteins which regulate cell-mediated immune responses. They are effector molecules that can instantly alter the quality of the immune response. The effect of a particular cytokine on a given cell depends on the cytokine, its extra cellular abundance, the presence (or absence) of the complementary receptor on the cell surface, and downstream signals activated by receptor binding [9]. As cytokines reflect the local or systemic inflammatory milieu, they could serve as biomarkers for potential clinical effect of the therapeutic interventions.

As cytokines act in networks, measurements of single cytokines is of limited value, emphasizing the need for simple, reliable, cost effective, and reproducible technology for the measurement of multiple cytokines. Several methodologies have been developed and employed for quantification of secreted cytokines. Immunoassays such as ELISA are currently the most commonly used techniques to quantify cytokines due to the high specificity and sensitivity [10]. Built on the same principle, more rapid, automated, and high throughput methods have been developed [11]. More recently a bead-based multiplex immunoassays (MIA) with the FlowMetrix (currently know as xMAP^tm ^technology, Luminex, Austin TX USA) has been increasingly used for detection of multiple cytokines in a single sample [12].

A number of parameters can affect adequate and reliable measurements of cytokine levels in biological specimens collected in a (multicenter) clinical trial including the timing of sampling, sample handling and storage, and even the choice of plasma or serum (various blood collection tubes). In some cases, such as inflammatory diseases, numerous endogenous plasma proteins such as heterophilic antibodies, soluble receptors, complement, immune complexes, lysosyme, collectins (lectins) and some acute phase proteins can also interfere with immunoassays such as MIA and ELISA [13].

We and others have previously shown that technical prerequisites for an "in-house" multiplex immunoassay have done comparison studies with ELISA's. In this study we set out to describe parameters which are critical for obtaining accurate cytokine measures from clinical samples, when using a multiplex cytokine detection platform, such as Luminex.

## Methods

### Serum and Plasma collection

Blood samples were collected from 4 healthy volunteers using the following blood collection tubes; normal clotting tube (SST II Advance, BD Biosciences) for serum and sodium heparin (NH), EDTA, or sodium citrate (NC) tubes for collecting plasma (all BD Biosciences) in the morning on 2 following days. All samples were kept on room temperature and were spun within 1 hour at 700 × g at room temperature. Cell free plasma or serum was aliquoted and stored at -80°C until analysis. Before analysis all thawed samples were centrifuged through a polypropylene centrifuge tube containing a 0.22 μm nylon membrane (Spin-X column; Corning, Corning, NY, USA) to remove debris. Non-specific heterophilic antibodies, such as natural polyspecfic antibodies, idiotypic antibodies and natural rheumatoid factors, were pre-absorbed, without loss of cytokine concentration, from all samples with protein-L pre coated ELISA plates (Pierce, Rockford, IL, USA) as described previously [14]. 100 μl of sample well was incubated for 1 hour at room temperature under continuous shaking. As this incubation step removes 60-80% of the total immunoglobulin fraction without depleting natural occurring cytokines [14]samples were then diluted with 10% v/v normal rat and mouse serum (1:1 ratio; Rockland, Gilbertsville PA, USA) and incubated for 10 additional minutes at room temperature to block any residual interfering proteins which are able to bind (a) specifically to rat or mouse immunoglobulins. Animal serum batches were tested upfront, and only used when they did not show any cross-reactivity within the assay.

All samples obtained were approved for collection by the medical ethical committee of the UMC Utrecht. Informed consent was obtained from each individual who donated samples.

### Cytokine induction in whole blood cultures

Cytokine production was induced using sodium heparin whole blood samples that were stimulated with a combination of 100 ng/ml lippopolysaccharide (LPS, Sigma Sigma-Aldrich, Zwijndrecht, the Netherlands) and 7 μg of phytohemagglutinin (PHA; Murex Biotech, Dartford, United Kingdom) per ml. Ten μl of the LPS/PHA cocktail was added to 1 ml whole blood and cultured for 6, 24 and 48 h at 37°C in 5% CO2. After culture the samples were centrifuged, and supernatants of all 3 time points were pooled and frozen at -80°C until further analyses by MIA

### Internal control sample

Sodium heparin blood samples were collected from 4 healthy volunteers and peripheral blood mononuclear cells (PBMC) were isolated by Ficoll Isopaque density gradient centrifugation (1.077 g/cm^3^; Amersham Pharmacia Biotech AB, Uppsala, Sweden). Cultures were performed in RPMI 1640 tissue culture medium supplemented with 100 U/ml penicillin-streptomycin, 2 mM L-glutamine and 10% heat inactivated fetal bovine serum (all Invitrogen, Gaithersburg, MD USA). We have shown previous that protein resources could interfere with cytokine assays [15]. FBS was tested upfront to show it did not induce cell activation/proliferation or cross reaction with human cytokines. Our batch of FBS was used for culturing PBMC's at different concentrations (5, 10 and 40%, during 96 hrs) and did not show any activation, measured by 3 H incorporation [16] or induction of cytokines by human PBMC measured by MIA (data available upon request). Cells were cultured in round bottom microtiter plates (Greiner, Alphen aan de Rijn, The Netherlands) at 2 × 10^5 ^cells per well and incubated for 24 to 48 hours at 37°C. A combination of 100 ng/ml LPS (Sigma-Aldrich) and 7 μg of PHA (Murex Biotech) per ml, was added to each well, after culture cell free supernatants were collected, pooled and frozen in 200 μl aliquots at -80°C. This sample was included in every MIA as an internal control (see below).

### Multiplex Immunoassay

The antibody pairs and recombinant proteins used were purchased from commercial sources as described previously [14]. Carboxylated polysterene microspheres were purchased from Bio-Rad Laboratories (Hercules CA, USA). Covalent coupling of the capture antibodies to the microspheres was performed as previously described [14,15]. Calibration curves from recombinant protein standards were prepared using two-fold dilution steps in serum diluent (R&D Systems, Abingdon, United Kingdom) as previously described [14]. Samples were measured and blank values were subtracted from all readings. All assays were carried out directly in a 96 well 1.2 μm filter plate (Millipore, Billerica, MA, USA) at room temperature and protected from light. A mixture containing 1000 microspheres per mediator (total volume 10 μl/well) was incubated together with a standard, sample or blank for 1 hour at room temperature. Next, 10 μl of a cocktail of biotinylated antibodies (16.5 μg/ml each) was added to each well and incubated for an additional 60 minutes. Beads were then washed with phosphate buffered saline (PBS) supplemented with 1% bovine serum albumin (BSA) and 0.5%-Tween 20 at pH of 7.4. After incubation for 10 minutes with 50 ng/well streptavidin R-phycoerythrin (BD Biosciences, San Diego CA, USA) and washing twice with PBS-1% BSA- 0.5%-Tween 20 pH 7.4 fluorescence intensity of the beads was measured in a final volume of 100 μl HPE-buffer (Sanquin Reagents, Amsterdam, the Netherlands). Measurements and data analysis of all assays were performed using the Bio-Plex system in combination with the Bio-Plex Manager software version 4.1 using five parametric curve fitting (Bio-Rad Laboratories). Performance characteristics of the multiple immunoassay, and biological variation in plasma samples of healthy controls are described elsewhere [14,15,17,18].

### Statistical analysis

All results are expressed as mean ± standard deviation (SD). A probability (p) less then 0.05 was considered significant different. For the calculation of the prolonged storage and stability of cytokines during freeze -thawing cycles, baseline values were set at 100% and follow-up points were related to this base value. All statistical analyses were performed using the statistical package for the social sciences (SPSS) software version 12.0.1 (SPSS, Chicago, IL, USA).

## Results

### Effect of anticoagulant on cytokine measurements

**Figure 1:**
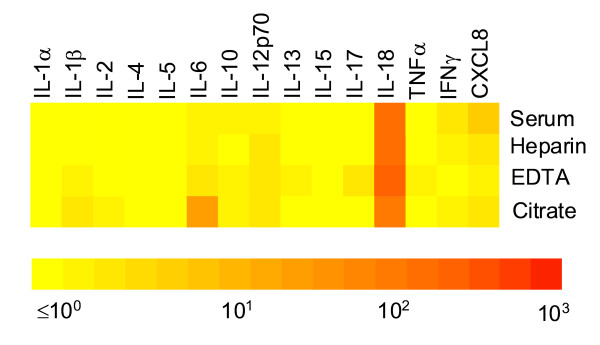
**Cytokine profiles in different blood collection tubes**. Blood samples were obtained from 4 healthy volunteers using various blood collection tubes after centrifugation cell free plasma (sodium heparin plasma, EDTA plasma and sodium citrate plasma) and serum cytokine levels were measured using a multiplex assay. Color profiles were generated using geometric mean values and plotted using a semi log scale as previously described [14].

To investigate potential effects of different blood anticoagulants on cytokine measurements by MIA, blood of 4 healthy volunteers was drawn in various tubes for serum, sodium heparin plasma, EDTA plasma and citrate plasma and cytokine profiles were measured. Obviously data analysis becomes complex when a large number of proteins are measured using a multiplex assay. Therefore we digitized our data creating color profiles of the protein spectrum as previously described[15]. Using this kind of representation protein fingerprints can be plotted as comparative data between different conditions or as quantitative data for an individual subject. As an example showing integral cytokine profiles the various blood drawing tubes. As expected, most cytokines were present in low or undetectable levels (figure 1). Only IL-18 was detectable and did not vary between the different sample collection tubes. In contrast IL-6 levels measured higher in citrate plasma whereas for CXCL8 highest concentrations were obtained from serum (figure 1).

As most cytokines are not expressed, individual person to person variation could only be calculated using IL-18 concentrations. Therefore blood samples were collected of the same individuals on 2 following days to study the variation between individuals using all various blood collecting tubes. Variations in IL-18 levels were similar for all different blood collection tubes and ranged between 6.1-9.8% depending on each individual.

Many studies have shown that various diseases have increased levels of cytokines in plasma or serum. As our healthy controls show no or low concentrations of cytokines we spiked the various plasma and serum samples of all donors with 1000 pg/ml of each cytokine to mimic increased concentrations of cytokines as shown in different diseases. This was done as we are in a pediatric setting and could not recruit any diseased individuals to test different blood tubes due to ethical restriction.

**Figure 2:**
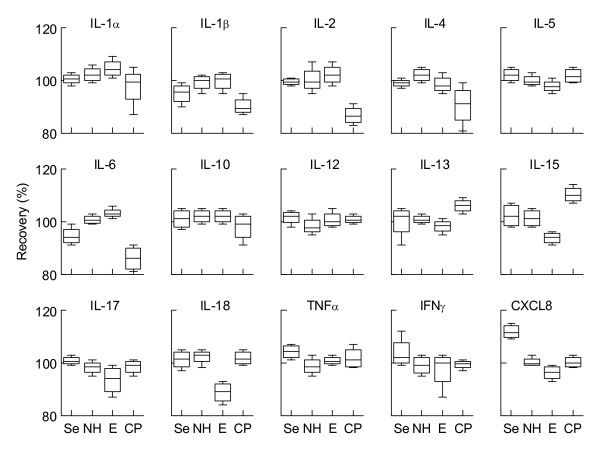
**Recovery of spiked cytokines in various types of plasma and serum**. Cytokines (1000 pg/ml) were spiked in various types of plasma and serum. Recoveries were measured using a multiplex immunoassay. Se serum, NH sodium heparin plasma, E EDTA plasma, CP sodium citrate plasma. Shown are means ± SD.

Recovery was measured in 2 assays on 2 consecutive days using a spiked sample, and a non-spiked sample as background. Overall recoveries of the spiked cytokines in the various serum and plasma samples ranged between 80 - 120% (figure 2). However, there were some marked inconstancies in recovery. In serum a significantly higher recovery for CXCL8 was found (113%, p < 0.05) whereas IL-6 levels were slightly lower in serum as compared to plasma samples. Sodium heparin and EDTA plasma showed a stable recovery off all cytokines, with the exception of IL-15 and IL-18 which was decreased in EDTA plasma (85% for IL-6, 87% for IL-18). The recovery of these two cytokines was significantly lower compared to the other samples (p < 0.05 in both cases). In citrate plasma lower recoveries were found for IL-1β, IL-2 and IL-6 (figure 2). Sodium heparin plasma showed the most constant recovery of all cytokines tested and therefore is the preferred anticoagulant.

### Stability of cytokines during long time storage

**Figure 3:**
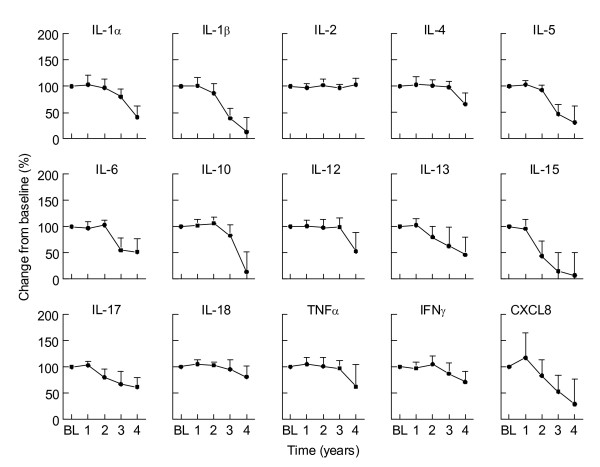
**Long time stability of induced cytokine profiles**. Sodium heparin whole blood from 3 individuals was stimulated with a combination of LPS and PHA to induce cytokine profiles. Samples were measured at baseline (BL) and at various time points. Baseline value was set at 100%. Shown are means ± SD.

In most clinical trials the inclusion period, intervention and follow-up periods run over a periods spanning several years. When the measurements are not performed directly, but at the end of the study, stability of cytokines has to be guaranteed during prolonged storage. As shown above, plasma from healthy individuals contains only low levels of most cytokines, sodium heparin whole blood from 4 individuals was stimulated with a combination of LPS and PHA which result in production of a wide array of cytokines. Cells were removed by centrifugation and samples were stored at -80°C. To determine cytokine degradation during long time storage, samples were measured directly (baseline) and at various time points afterwards (up to 4 years). As shown in figure 3 most cytokines are stable for up to two years. Degradation of cytokines is observed for IL-13, IL-15, IL-17 and CXCL8 already within one year of storage, whereas IL-2, IL-4, IL-12 and IL-18 are stable for up to 3 years. Other cytokines, such as IL-1α, IL-1β, IL-5, IL-6, and IL-10 degraded up to 50% or less of baseline values within 2-3 years of storage (figure 3).

### Cytokine stability upon freeze - thawing

**Figure 4:**
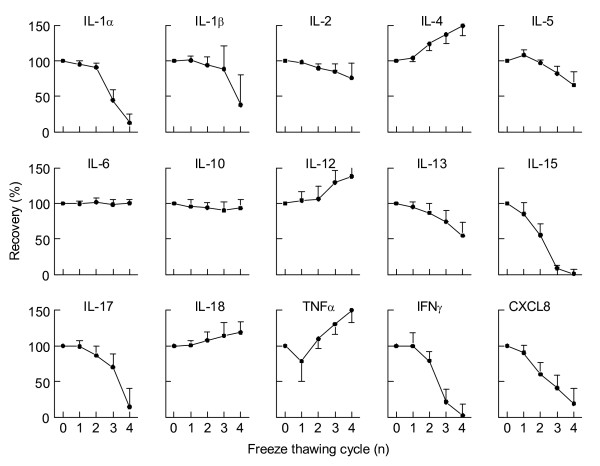
**Recovery of cytokines after multiple freeze - thawing cycle**. Aliquots of the same LPS/PHA stimulated samples were freshly thawed or underwent multiple freeze - thawing cycles and cytokines were measured simultaneously. Shown are means ± SD.

Next we questioned how stability of cytokines is influenced by freeze thawing cycles. Therefore a cytokine profile was induced by stimulation of sodium heparin whole blood as described above. From the same samples, aliquots were subjected to multiple freeze - thawing cycles and cytokines were measured. IL-6, and IL-10 are stable throughout multiple freeze thawing cycles, whereas IL-4, IL-13, IL-15, IL-17, TNFα, IFNγ and CXCL8 levels either rise (IL-4 and TNFα) or drop (IL-13, IL-15, IL-17, IFNγ and CXCL8) after one or more freeze - thawing cycles (figure 4). These data indicate that samples for cytokine measurements should not have been subjected to repeated freeze-thaw cycles.

### Quality control for multiplex immunoassay

**Figure 5:**
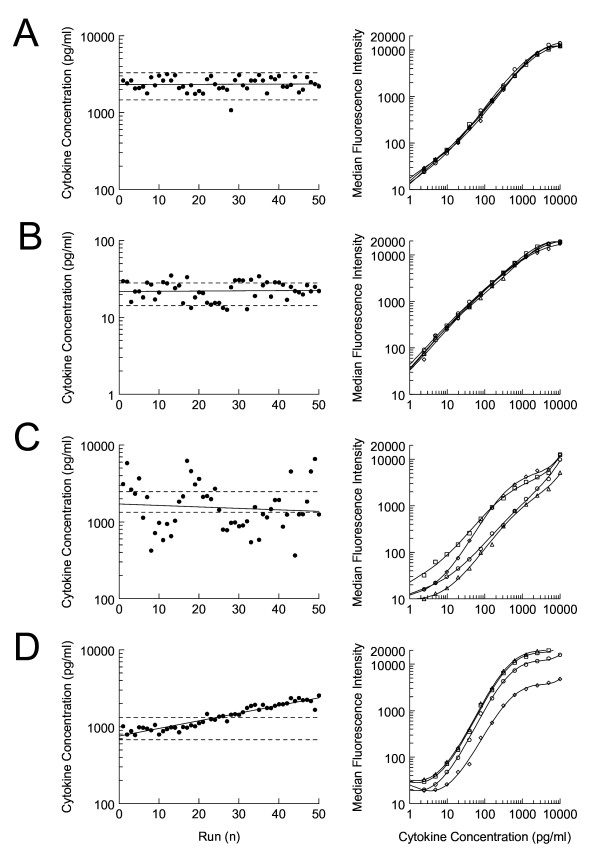
**Longitudinal assay performance using an internal control sample**. Left panel shows variability in time of observed cytokine values of the internal control sample for IL-1β (A), IL-4 (B), I-L6 (C) and TNFα (D) in 50 consecutive runs covering a period of 12 months. Right-hand panel shows overlays of 4 separate standard curves of the corresponding cytokines.

As shown above, cytokines degrade at -80°C during long time storage, such that samples should be measured with a timely interval using a robust, reliable, and consistent assay. Therefore it is essential to implement a QC system for multiplex immunoassays. When an in-house assay is developed for a bead based MIA, all the different steps of the assay must be monitored for the use with samples from clinical trials. Coupling of primary antibodies to the microspheres is rather straightforward and QC assessment of this procedure is previously described[15]. However, an internal control sample is needed to monitor several other parameters of the assay. We measured our internal control sample undiluted and diluted 1:100 with HPE buffer over a time period of 1 year to test for consistency. Intra-assay variation was <7% and inter-assay variation ranged between 5 and 20% (data not shown). We set our cut off value on the 2-SD range of the mean measurement of this sample run repeatedly over 20 different days. The range was chosen since cytokine concentrations of biological control samples will be either at the lower or upper end of the standard curve. However, due to this approach, the intra-assay CV of a low expressed cytokine will therefore more frequently fall outside the 2-SD boundaries then a high expressed cytokine. Intra-assay variation is higher for cytokines present in relative low concentrations as compared with a cytokine which is abundantly expressed an internal control sample (figure 5A/B).

Inter-assay variation will also be high in case of poor antibody performance. This phenomenon can be easily detected by comparison of the relevant standard curves. Normally, inter-assay comparison of standard curves will show overlapping or parallel curves (figure 5A and 5B) but when the detection and/or matching catching antibodies perform poorly this will result in dispersed graph pattern with variable slopes (figure 5C).

Finally, when it is acknowledged that cytokines in plasma samples can deteriorate over time, even when stored at -80°C, the same hold true for the recombinant proteins used for generating standard curves. When such degradation occurs, the standard curves will shift to lower fluorescence intensity. This will result in apparent higher values of the internal control samples as wells as higher cytokines concentrations of clinical samples when similar concentrations recombinant proteins are used for generating standard curves (figure 5D).

## Discussion

Cytokines are involved in the effector phase of all inflammatory diseases, and thus, could serve both as biomarkers for disease severity and as targets for therapy. Anti cytokine therapy was first introduced in the early eighties of the last century, with the first successful application of an anti cytokine therapy trial involving anti-IFN-alpha antibodies in patients with Rheumatoid Arthritis [19]. Since then, several monoclonal antibodies against cytokines or cytokine receptors as well as other immune modulating drugs are used for a variety of diseases [1-6].

Because of the interconnectivity of immunoregulatory circuits, blocking or activation of specific components of the immune system can result in major changes in the cytokine milieu, which may lead to unexpected side effects. As an example, the development of systemic lupus erythematosus-like symptoms during anti TNFα therapy in Rheumatoid Arthritis patients is due to accumulation of another cytokine, IFN-α, a direct consequence of the TNFα blockade [20,21]. Careful monitoring of cytokines is necessary to assess the impact of drugs that influence the immune response, particularly during early phase clinical trials. As multiple cytokines may be effected, techniques for measuring panels of cytokines are of most value.

The microsphere-based multiplex immunoassay measurement allows the simultaneous analysis of multiple secreted cytokines in a variety of bodily fluids [12]. This technology can detect up to 100 different mediators using as little as a 50 μl sample volume [11,22,23], making this technology very useful in clinical trials, especially when volumes are limited.

For a multiplex assay to perform adequately the conditions should be optimal for every individual component. However, many clinical trial designs underestimate the impact of parameters which can impact reliable cytokine measurements such as sample handling and storage, and quality control of the platform used for cytokine detection.

First, timing of sampling is important. Cytokines are under neuroendocrine control and thus have a diurnal rhythm [24,25]. Many pro-inflammatory cytokines, such as IL-1, IL-6, TNFα and IFNγ are linked to melatonin and peak early in the morning [26]. Rheumatoid arthritis patients have increased IL-6 levels in the morning which correlate with clinical symptoms [27,28]. Besides diurnal rhythms, exercise also impacts cytokine levels in blood, as after exercise, among others, muscle cells release IL-6 and which will result in temporarily elevated cytokine levels [29,30].

Secondly, proper sample handling and storage is critical for reliable measurement of circulating cytokines. When sampling takes place plasma and serum should be separated as soon as possible. In this study we kept our samples at room temperature and froze plasma and serum sample at -80°C within 1 hour after blood draw. A delay of sample processing containing cellular components will lead to different cytokine expression profiles by either degradation, absorption, or cellular production of cytokines [31]. Furthermore, a careful consideration should be made for the use of blood collection tubes during trial design. During clotting or by centrifugation, platelets can release various cytokines such as IL-6 and CXCL8 [32]. Furthermore cytokine binding to, or release from their soluble receptors can result in an under- or overestimation during spiking assays. For instances specific binding and release of IL-1, IL-6, and IL-18 towards their soluble receptor is well documented [33,34]. This phenomenon can at least partly account for the large variation found in our spiking assays (figure 2). We show here that the use of various blood collection tubes significantly affects cytokine measurement, with sodium heparin tubes showing most consistent cytokine recovery.

Cytokines should be stored below -80°C for longer time. Although degradation will occur also at this temperature after longer periods of storage (figure 3), sample storage at temperatures of -20°C degradation occurs at faster pace [35]. Furthermore multiple freeze - thawing cycles also affect cytokine recovery in clinical samples. Although most cytokines are stable in a high protein matrix such as plasma, during the first freeze - thawing cycle an additional freeze - thawing cycle should be avoided at any time (figure 4).

The third critical factor is the quality control of the platform used. Obviously, assay performance is strongly dependent on the quantity of antibodies used as well matrix effects. We and others showed that quality control of different components (e.g. recombinant proteins, sample diluents, microsphere coupling etc.) of the multiplex immunoassay can be performed and implemented easily in a in-house assay [15,22,36]. In commercially available kits internal QC samples are mostly lacking. However some commercial kits suggest to use several dilutions of the recombinant standard curves as an spiked in QC sample [37]. Though, the amount of recombinant standards and diluents for construction of calibration curves are sufficient for a single kit and can not be used for long-term monitoring of performance [38]. With the use of an in-house assay, standard curves can either be constructed with or calibrated on NIBSC/WHO standards. However, when comparing different commercial assays using these NIBSC/WHO standards, large differences in observed cytokine concentrations are found [39,40]. Use of different antibodies (clones) is only part of the explanation for this phenomenon. Other, more likely culprits are matrix effects, e.g. because of heterophilic antibodies that are often present in human inflammatory diseases [41]. We have shown previously that removing antibodies with protein-L followed by blocking with 10% rat/mouse serum is sufficient to reduce such matrix effects [14] where others demonstrated that blocking with fetal bovine serum (FBS), was effective in only a minority of the samples [42]. Since FBS does not contain immunoglobulins, this underscores the possibility that proteins, other than antibodies, can interfere with the assay. Other matrix effects, such as protein content and pH can also influence the assay performances and should be standardized when using this assay for clinical trial design [11,43]. For samples with high protein content such as plasma or serum most commercial assays require dilutions for ideal assay performance, while an in-house assay condition can be set in such a way that samples do not have to be diluted.

The necessity of sample dilution is a major drawback, since it proportionally reduces assay sensitivity. This has major impactions for detection of cytokines such as IL-4, which is already biological active in the lower pg/ml range. Another disadvantage for dilution is that cytokines can be bound to their soluble receptors or binding proteins, resulting in biological non-active cytokines. This binding can, at least partly, be reversed when dilutions are applied, thus resulting in over estimation of the true cytokine concentration [34,44].

However, previous studies have shown that without addressing matrix effects in undiluted samples false positive findings are found in up to 90% of healthy individuals which could be reversed by incubating with rodent serum [22]. However blocking with rodent serum alone is not sufficient to remove false positive values from patients which are positive for auto antibodies such as rheumatoid factor [11]. Therefore in this study, we applied a relative small dilution (10 v/v %) with rodent serum after protein-L incubation to avoid a-specific binding of matrix proteins.

All points described concerning the biological properties of cytokines, such as long time stability and freeze thaw cycles are not only relevant multiplex immunoassays, but also be applied to other technology used for cytokine measurements in a clinical (trial) setting. Multiplex immunoassays have many advantages for biomarker development in clinical settings. It can measure multiple mediators at once and has the flexibility to easily add any new target protein to the assay. This study underlines that multiplex immunoassays have many advantages for the use in clinical trials, provided that a strict quality control system is in place.

## Conclusion

In this study we set out to investigate several processes that might impact cytokine profiles such as blood collecting tubes, duration of storage, and number of freeze thawing cycles. Concluding from the data we present here in this study we suggest to process clinical sample as soon as possible (within 1 hour), and store them at -80°C not longer then 2 years for cytokine analysis without any freeze - thawing cycle. Furthermore we recommend preparing an internal control sample which is applicable for the samples of interest, which is kept under similar conditions as clinical sample (stored at -80°C, not longer then 2 years, without freeze -thawing cycles). Single use aliquots of this sample can be a freshly prepare as quality control sample for each assay.

In the context of clinical trial design in which cytokine measurements play a role, these apparently trivial underappreciated processes described here can greatly impact the reliability and reproducibility cytokine measurements and should be taken into account when designing future clinical trials.

## Authors' contributions

WdJ performed research and analyzed data together with KB and GTR. BJP and VSM supervised the study. WdJ, BJP and VSM wrote the article. All authors had full access to all data, read and approved the final manuscript.
